# Glioblastoma Immunotherapy Targeting the Innate Immune Checkpoint CD47-SIRPα Axis

**DOI:** 10.3389/fimmu.2020.593219

**Published:** 2020-11-27

**Authors:** Jinyang Hu, Qungen Xiao, Minhai Dong, Dongsheng Guo, Xudong Wu, Baofeng Wang

**Affiliations:** ^1^ Department of Neurosurgery, Tongji Hospital, Tongji Medical College, Huazhong University of Science and Technology, Wuhan, China; ^2^ Department of Cell Biology, 2011 Collaborative Innovation Center of Tianjin for Medical Epigenetics, Tianjin Key Laboratory of Medical Epigenetics, Tianjin Medical University, Tianjin, China; ^3^ Department of Neurosurgery, Tianjin Medical University General Hospital and Laboratory of Neuro-Oncology, Tianjin Neurological Institute, Tianjin, China

**Keywords:** glioblastoma, immune checkpoint, CD47-SIRPα, tumor-associated macrophages/microglia, glioblastoma microenvironment

## Abstract

Glioblastoma Multiforme (GBM) is the most common and aggressive form of intracranial tumors with poor prognosis. In recent years, tumor immunotherapy has been an attractive strategy for a variety of tumors. Currently, most immunotherapies take advantage of the adaptive anti-tumor immunity, such as cytotoxic T cells. However, the predominant accumulation of tumor-associated microglia/macrophages (TAMs) results in limited success of these strategies in the glioblastoma. To improve the immunotherapeutic efficacy for GBM, it is detrimental to understand the role of TAM in glioblastoma immunosuppressive microenvironment. In this review, we will discuss the roles of CD47-SIRPα axis in TAMs infiltration and activities and the promising effects of targeting this axis on the activation of both innate and adaptive antitumor immunity in glioblastoma.

## Introduction

Glioblastoma (GBM) is the most common primary malignant brain tumor in adults and is characterized by invasive growth and frequent recurrence. Despite of advances in surgical resection, radiotherapy, and chemotherapy, the median survival time of patients is only 12 to 15 months; the 3-year survival rate is approximately 10% (1, 2). Great progress has been made in the development of immunotherapy for extracranial tumors. However, most clinical trials of immunotherapy for GBM have shown only a moderate response and no significant improvement in over survival (OS) (3).

**Figure 1:**
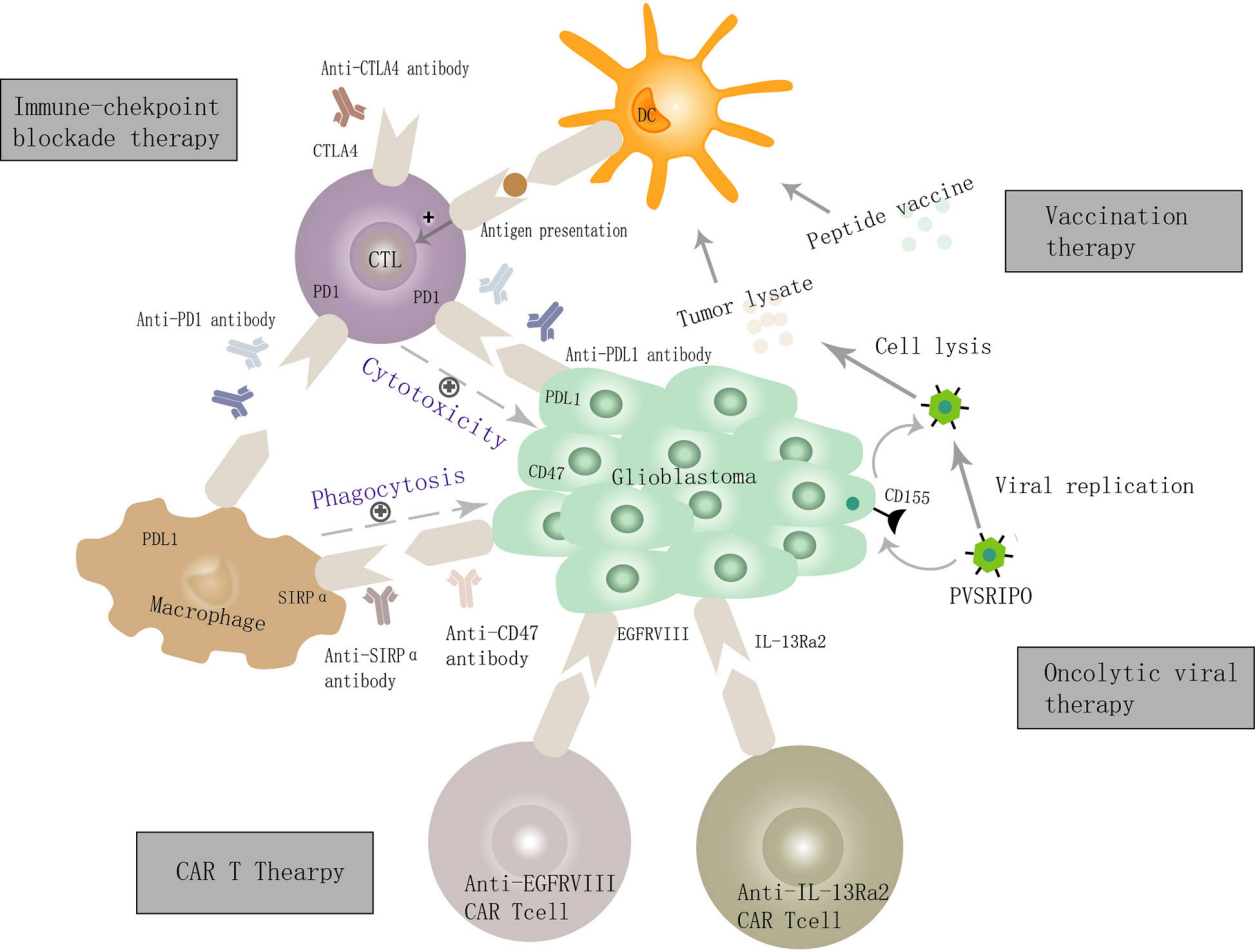
Cellular and molecular mechanisms of GBM immunotherapy. GBM cells overexpress PDL1, CD47, and other immunosuppressive molecules and bind the ligands present on cytotoxic T lymphocytes (CTLs) and macrophage, and thereby inhibit the innate and adaptive immune function, leading to the immune escape of GBM. Targeting immune checkpoint molecules such as PDL1, CD47, and CTLA4 can activate both innate and adaptive anti-tumor immunity. The mechanism of oncolytic virus therapy is mainly *via* the creation of viruses that can selectively infect GBM cells, defeat GBM cells, and enhance adaptive anti-tumor immune responses by the dendritic cell and CTL. Several tumor-related antigens (e.g., IL-13Ra2, EGFRvIII) are expressed on the surface of GBM cells and are used as specific targets for (CAR) T cell therapy to achieve a precise treatment objective. The vaccination strategy mainly mediates the activation of CTLs by antigen-presenting cells, thus killing GBM cells.

Currently, immunotherapy for GBM includes immune checkpoint blockade therapy, vaccination therapy, oncolytic virus therapy, and CAR-T therapy (4–6), which mainly take advantage of the adaptive anti-tumor immunity (**Figure 1**). Accumulating evidence suggests that the GBM microenvironment is characterized by high myeloid cell content, relatively few tumor-infiltrating lymphocytes (TILs) (7, 8)and T cell dysfunction (9). In contrast, tumor-associated microglia/macrophages (TAMs) account for 30% to 40% in GBM (10, 11). Approximately 85% of them are bone marrow-derived infiltrating macrophages/monocytes while the remaining fractions are locally resident microglia (12, 13), which engage in reciprocal interactions with GBM and adaptive immune cell to mediate tumor immune escape (14–16), promote tumor growth and progression (17–21). Therefore, reeducating, reactivating, and reconstructing the TAMs functions in GBM immunosuppressive microenvironment makes them superior again is a promising field.

The strategies targeting TAMs fall into three main groups: 1) inhibiting recruitment of the bone marrow-derived infiltrating macrophages/monocytes (22–24); 2) promoting phagocytosis of tumor cells by TAMs and restoring its innate antitumor immunity (25, 26); 3) reprogramming TAMs to antitumor macrophages/microglial either directly through tumor cell killing or by reactivating adaptive antitumor immunity (27–30). The CD47-SIRPα Axis is currently the most widely studied innate immune checkpoint (31). Interestingly, the accumulating data shows that target the CD47- SIRPα axis bridging innate and adaptive antitumor immunity (15, 32). Targeting the CD47- SIRPα axis activates both innate and adaptive antitumor immunity (33), which is promising for GBM therapies. This review will discuss in more detail about the structure and regulation of innate immune checkpoint CD47-SIRPα and their functions in the immune-suppressive microenvironment and therapeutic potential in GBM. We would like to raise awareness of immune parameters in clinical stratification schemes and encourage discussions and improvements about innate anti-tumor immunity-oriented immunotherapies.

## Structure of CD47-SIRPα

The CD47 gene is located on chromosome 3q13 and encodes an integrin-associated protein. CD47 is an important “self-labeling” molecule in the immunoglobulin superfamily that contains an immunoglobulin variable-like amino-terminal domain, five transmembrane domains, and one carboxy-terminal intracellular tail (34, 35). Signal regulatory proteins (SIRPs) are inhibitory immune receptors encoded by a cluster of genes on chromosome 20p13, including SIRPα, SIRPβ1, SIRPγ, SIRPβ2, and SIRPδ (36). SIRPα binds to CD47 with high-affinity (37). Structurally, the extracellular domain of SIRPα consists of three immunoglobulins (Ig)-like domains (the NH2-terminal V-like domain and two C1 domains), a single transmembrane segment, and the intracellular segment containing four tyrosine residues that form two typical immune-receptor tyrosine-based inhibition motifs (ITIMs). When CD47 expressed on the surface of GBM cells binds to the NH2-terminal V-like domain of SIRPα on myeloid cells, phosphorylation of the tyrosine residue in the ITIM motif results in the recruitment and activation of tyrosine phosphatase SHP1/SHP2. This process affects the levels of downstream de-phosphorylated molecules and inhibits the phagocytosis of GBM cells by macrophages (38). Hence CD47 serves as a critical “do not eat me” signal. However, the signaling mechanisms upstream and downstream of the CD47-SIRPα axis are incompletely understood.

## Expression and Regulation of CD47-SIRPα AXIS

CD47 has been found to be highly expressed in GBM cells, especially glioblastoma stem cells (39). Its expression levels are positively correlated with glioma grade and are associated with worse clinical outcomes (39–41). Hence It has been regarded as a critical biomarker for glioblastoma (42). Amounting studies have demonstrated that MYC (43), PKM2-β-catenin-BRG1-TCF4 complex (44), NF-Kβ (45), and NRF1 (46) may bind at the promoter of CD47 to regulate its transcription. SIRPα is expressed on myeloid cells, including macrophages, dendritic cells (DCs), neutrophils, and nerve cells (neurons, microglia) (36). Interestingly, SIRPγ is expressed on human activated T cells and also binds to CD47, albeit with a lower affinity than SIRPa (31), which may also play a pivotal role in the adaptive antitumor immunity. More comprehensive research into the dynamic control of the CD47-SIRP axis will be greatly helpful for us to understand its functions and optimize its targeting strategies.

## The Functions of The CD47-SIRPα AXIS in Glioblastoma

The exact functions of CD47 in GBM are still in debate. The increased expression of CD47 were found to promote the proliferation and invasion of GBM cells while it did not affect the proliferation ability of normal astrocytes (47, 48). However, some other studies found that CD47 could enhance the invasion ability of GBM cells through the PI3K/AKT pathway but had no effect on proliferation (49). Moreover, CD47 positive GBM cells possessed many characteristics that associate with cancer stem cells, which implies worse clinical outcomes (50). Accumulating evidence suggests that CD47 binds SIRPα on macrophages, neutrophils, and dendritic cells, subsequently inhibiting the cytotoxicity of macrophages and neutrophils, limiting the antigen-presenting function of dendritic cells, and inhibiting both innate and adaptive immune functions (38, 50, 51).

## The Significance of Targeting CD47-SIRPα AXIS in The GBM Microenvironment

**Figure 2:**
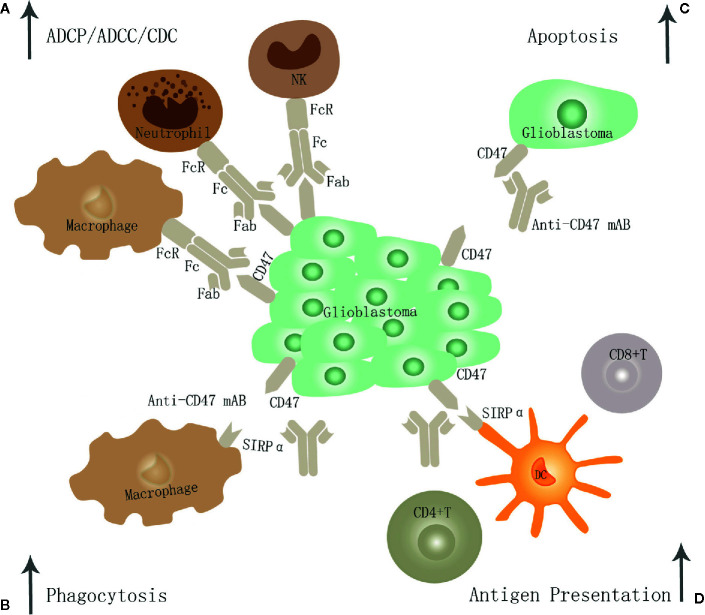
The potential mechanism of CD47-SIRPα inhibition in GBM. Targeting the CD47-SIRPα axis may exert anti-GBM effects through the following four pathways: **(A)** Eliminate GBM cells through traditional antibody Fc-dependent mechanisms, including ADCP, ADCC, and CDC. **(B)** it leads to enhanced tumor cell phagocytosis by macrophage through disrupting the binding of CD47 to SIRPα. **(C)** Promote apoptosis of GBM cells. **(D)** Restore dendritic cells' function to present antigen to CD4+ and CD8+T cells, thereby stimulating an anti-tumor adaptive immune response.

Targeting the innate immune checkpoint CD47-SIRPα axis enhances the phagocytosis rate, resulting in a significant survival benefit even in the absence of peripheral macrophages (52). Therefore, when studying the effects of CD47-SIRPα immunological checkpoint inhibitors on the phagocytic function of macrophages *in vitro*, their impact on microglia function must be considered. Targeting the innate immunity checkpoint CD47-SIRPα axis exerts anti-GBM efficacy mainly through the following four pathways (**Figure 2**).

In the first pathway, it leads to enhanced tumor cell phagocytosis by both M1 and M2 macrophage subtypes and shifts the phenotype of macrophages toward the M1 subtype *in vivo* (53). And the phagocytic potential of M1 was similar to that of M2 *in vitro*. Phagocytosis by M1 increased in a CD47-dependent manner by the neutralizing antibody and siRNA against CD47 but not in M2 (54). In line with previous studies, Zhu et al. suggest that surgical resection combined with anti-CD47 immunotherapy was shown to promote the recruitment of macrophages and promote phagocytosis of glioblastoma (25). Li et al. come to a similar conclusion that humanized CD47 antibody HU5F9-G4 inhibits CD47 expression, enhanced tumor cell phagocytosis by macrophage, improves the survival time of animals, and has nontoxic effects on neurons and other tissues in a xenograft model derived from the malignant brain tumor (50).

In the second pathway, it enhances the antigen presentation ability of DC to generate potent T-cell priming and adaptive antitumor immune responses (32, 33). Christina et al. suggest that anti-CD47 treatment alone has limited anti-tumor effects and is inefficient in inducing changes within the tumor immune microenvironment or eradicating murine GBMs in immune-competent hosts. Instead, combined TMZ and CD47 blockade activates the cGAS-STING pathway, increases T-cell priming, and thereby activates both innate and adaptive immune responses *in vivo*. Hence the combination treatment is further augmented by adjuvant PD-1 blockade (33). In addition, radiotherapy was demonstrated to enhance the anti-CD47 therapeutic effects (55).

In the third pathway, glioblastoma cells may be eliminated *via* traditional antibody Fc-dependent mechanisms, including neutrophil cell-mediated antibody-dependent cellular cytotoxicity (ADCC) and macrophage-mediated antibody-dependent cellular phagocytosis (ADCP) (56, 57). Recent studies have demonstrated that neutrophil ADCC toward cancer cells occurs through a mechanism called trogocytosis, which can be further improved by targeting CD47-SIRPα interactions (58). The bispecific antibodies targeting the membrane-proximal epitope of MSLN improve ADCC activity by augmenting FcγR-IIIA activation and enhanced ADCP *via* a more efficient blockade of the CD47/SIRPα axis (59).

In the fourth pathway, it can induce apoptosis of tumor cells directly (60). It has been shown that CD47 antibody-induced apoptosis of cancer cells is due to neither ADCC nor CDC. Instead, such antitumor activity by bivalent scFv is presumably attributable to cell death caused by the ligation of CD47 (61, 62). And tumor cells may be eliminated through direct induction of apoptosis by a novel pathway involving regulation of cAMP levels by heterotrimeric Gi with subsequent effects mediated by PKA (63, 64). However, its specific functions and mechanism in GBM require further studies.

Collectively, targeting the immune checkpoint complex CD47-SIRPα has been shown as a promising anti-tumor strategy that may remodel the GBM microenvironment, restore innate and adaptive immunity functions, and improve the prognosis of patients with GBM. Notably, these promising strategies still need considerable refinement before becoming the standard clinical treatment options for GBM.

## Immunological Checkpoint Inhibitors Targeting CD47-SIRPα Axis

Currently, inhibitors targeting CD47-SIRPα immunological checkpoints are in preclinical and clinical study phases. These inhibitors include 1) monoclonal antibodies (CD47 monoclonal antibody Hu5F9-G4, human IgG4 subclass; SIRPα monoclonal antibody FSI-189), which are mainly to block the anti-phagocytosis signal and reactive macrophages to attack and destroy tumor cells (65, 66); 2) recombinant fusion proteins (TTI-621, SIRPα-Fc fusion protein, human IgG1 subclass; TTI-622, SIRPα-Fc fusion protein, human IgG4 subclass), which are composed of the N-terminal V domain of human SIRPα and the human IgG Fc region. The N-terminal V domain of human SIRPα bind human CD47 on tumor cells and prevent it from delivering inhibitory signals to macrophages. At the same time, The IgG Fc region of SIRPαFc can bind to the high-affinity receptor FcγRI (CD64) as well as to the low-affinity receptors FcγRII (CD32) and FcγRIII (CD16) on macrophages to further enhance macrophage-mediated ADCP, tumor antigen presentation, and effective anti-tumor activity. Lower affinities for normal red blood cells and reduced side effects are important advantages of recombinant fusion protein therapies (67); 3) bispecific antibodies (NI-1701, anti-CD19/anti-CD47 bispecific antibody; NI-1801, anti-CD47/mesothelin bispecific antibody); VEGFR1D2-SIRPαD1. NI-1701 has three arms. The targeting arm binds CD19, a cell-surface antigen expressed by B-cell-origin tumors. The effector’s arm destroys the CD47-mediated anti-phagocytosis signal. The Fc arm of the antibody can recruit macrophages and other innate immune killer cells. NI-1801 destroy mesothelin-positive solid tumors through the innate immune system; VEGFR1D2-SIRPαD1 consists of the second extracellular domain of VEGFR1 (VEGFR1D2) and the first extracellular domain of SIRPα (SIRPαD1), which exerted potent anti-tumor effects *via* suppressing VEGF-induced angiogenesis and activating macrophage-mediated phagocytosis (68–70). Among the immunological checkpoint inhibitors, Hu5F9-G4, TTI-621, and TTI-622 are undergoing Phase I clinical trials, although the complete data have not been published (71).

## Safety Assessment and Future Perspectives

The main concern of CD47 inhibitors is the risk of hematological toxicity such as anemia, thrombocytopenia, and leukopenia, given the high expression of CD47 on normal red blood cells and platelets (72, 73). Preclinical studies show that CD47 inhibitors in mice are well-tolerated, with no obvious signs of toxicity (50, 74). However, Arch Oncology and Celgene discontinued a clinical trial of the CD47 inhibitors because of possible off-target effects such as anemia (75). One of the most important issues is to reduce or avoid potential toxicity while preserving anti-tumor effects.

The toxicity of anti-CD47/SIRPα antibodies appears to be Fc-dependent. It may be desirable to block the SIRPα-CD47 interaction by antibodies devoid of the Fc portion or optimize the structure of the Fc portion. Meanwhile, targeting tumor cells for FcR-mediated phagocytosis using intact antibodies (31). For example, the macrophage checkpoint inhibitor 5F9 combined with rituximab showed promising activity in patients with aggressive and indolent lymphoma, with no clinically significant toxicity (65). SIRPα expression in normal cells is much narrower than CD47 and its targeting may result in more limited toxicity, such as recombinant fusion proteins TTI-621 and ALX148 and high-affinity monomeric SIRPα with lower affinities for normal red blood cells (67, 76, 77), which is also an ideal strategy. Red blood cells act as a “sink” binding to anti-CD47 antibodies and reduce the effective therapeutic dose. Hence, optimized initiation dose and maintenance dose to achieve an effective therapeutic blockade of CD47/SIRPα Axis is pivotal. For example, a non-human primate study revealed that the effector function competent mAb IgG1 C47B222-(CHO) showed antitumor activity *in vitro* and *in vivo* while decreased red blood cells (RBC), hematocrit and hemoglobin by >40% at 1 mg/kg (78). However, toxicokinetic studies suggest that alternative treatment regimens for Hu5F9-G4 (a low initiation dose and a higher maintenance dose) may contribute to achieving therapeutic efficacy with lower toxicity (71).

## Conclusions

Preclinical studies have found that targeting the immunological checkpoint complex CD47-SIRPα can inhibit the development of glioblastoma, enhance the function of phagocytic cells, restore the function of dendritic cells and T lymphocytes, and exert anti-tumor effects by improving innate and adaptive immune responses. However, there are still a series of biosafety problems such as anemia that remain to be solved. Besides, it is incompletely understood how CD47-SIRPα blockade works at the molecular level. Further understanding of the mechanism of CD47-SIRPα inhibitors will help to improve the efficacy and reduce the side effects. Ongoing clinical trials will further clarify their efficacies as single agents or in combination therapies. Careful observations of cytotoxic T cell response, T cell exhaustion, immune gene expression signatures in GBM subtypes, immune suppression (predominant immunosuppressive cells such as TAMs) may aid in identifying patients suitable for this therapy, avoiding potential toxicities and designing optimal combination therapies.

## Author Contributions

HJ collected the literature and drafted the manuscript. DM modified the paper format, and WB, WX, DG, and QX guided the writing and made significant revisions to the manuscript. All authors contributed to the article and approved the submitted version.

## Funding

This work was supported by the National Natural Science Foundation of China (No. 81472364, 81702480, 81874086).

## Conflict of Interest

The authors declare that the research was conducted in the absence of any commercial or financial relationships that could be construed as a potential conflict of interest.
